# Porous Hydrogels Prepared by Two-Step Gelation Method for Bone Regeneration

**DOI:** 10.3390/jfb16030100

**Published:** 2025-03-13

**Authors:** Yongzhi Li, Jiangshan Liu, Jiawei Wei, Li Yuan, Jiaxin Hu, Siluo Dai, Yubao Li, Jidong Li

**Affiliations:** Research Center for Nano-Biomaterials Analytical and Testing Center, Sichuan University, Chengdu 610065, Chinadaisiluo@163.com (S.D.);

**Keywords:** porous hydrogels, double crosslink, vascularized bone regeneration, calvarial defect repair

## Abstract

Hierarchical porous hydrogels possess advantageous characteristics that facilitate cell adhesion, promote tissue growth, and enhance angiogenesis and osteogenesis. In this study, porous composite hydrogels were successfully prepared by a two-step gelation method with sodium alginate (SA), gelatin (GEL), and calcium hydrogen phosphate (DCP) as the main components. The fabricated porous hydrogels initially featured small pores (approximately 60 μm), and gradually evolved to large pores (exceeding 250 μm) during the gradual degradation in the cellular microenvironment. In vitro cell culture experiments indicated that these hydrogels could enhance the proliferation and osteogenic differentiation of bone marrow mesenchymal stem cells due to the hierarchical porous structure and the incorporation of DCP. Subcutaneous implantation and cranial defect repair experiments in Sprague−Dawley rats further confirmed that the small initial pore size of hydrogel scaffolds can provide more sites for cell adhesion. Additionally, the gradual degradation to form large pores was conducive to cell/tissue growth and blood vessel formation, ultimately being beneficial for vascularized bone regeneration. In summary, this study proposes an innovative strategy for developing porous hydrogels with gradual degradation for functional bone regeneration.

## 1. Introduction

As a highly hydrated functional material, hydrogel scaffolds can be customized via various fabrication techniques for biomedical applications [1,2,3,4]. Owing to their excellent biocompatibility, structural adaptability, functional diversity, and adjustable mechanical properties, these scaffolds have been widely utilized in the repair of critical bone defects [5]. However, the dense network of the hydrogel structure presents several challenges: (1) it restricts nutrient exchange and cell migration, hindering cell proliferation; (2) it exhibits limited vascularization capability; and (3) the degradation rate of dense hydrogels may not synchronize with the rate of bone regeneration [6,7,8]. Studies have demonstrated that hydrogel scaffolds with porous structures exhibit significant advantages in promoting cell migration, facilitating nutrient and waste exchange, enhancing vascular growth, and supporting tissue development [9,10,11,12]. In the cellular microenvironment, smaller pores (80–120 μm) promote cell infiltration and proliferation during the inflammatory stage, while larger pores (200–300 μm) facilitate nutrient exchange, oxygen diffusion, and vascularization during the tissue regeneration phase [13,14,15,16,17]. Furthermore, the development of a vascular network ensures the delivery of sufficient oxygen, nutrients, and factors that promote bone regeneration and functional reconstruction [18,19]. In recent years, techniques such as cyclic freeze–thaw, sacrificial templating, and 3D printing have been widely adopted for the preparation of porous hydrogels [20,21]. Nevertheless, the hydrogels prepared using these strategies exhibit a relatively defined pore size, which fails to strike an optimal balance between promoting early cell adhesion and facilitating subsequent tissue growth and angiogenesis. By integrating traditional “Egg-box” structures with phase separation techniques, it is anticipated that porous architecture hydrogels with gradual degradation can be developed, potentially addressing this challenge.

Sodium alginate (SA) is a linear anionic polymer composed of mannuronic acid (M) and guluronic acid (G) residues [22]. The G blocks within SA can form a characteristic “Egg-box” structure with divalent cations such as Ca^2+^ and Zn^2+^ [23,24,25]. Therefore, SA has been widely utilized to prepare porous hydrogels [26,27]. Nevertheless, the use of soluble calcium salts (CaCl_2_) as crosslinking agents via external gelation often results in hydrogels with non-uniform and low porosity [28,29,30,31]. To address this issue, researchers have explored the internal gelation method, employing low-solubility calcium salts (CaCO_3_) combined with acidifiers like Glucono-δ-Lactone (GDL) [32]. GDL serves as an acidifier that gradually hydrolyzes to release acid, thereby promoting the gradual release of Ca^2+^ from low-solubility calcium salts. This slow-release mechanism allows Ca^2+^ to permeate through the sodium alginate matrix from the inside out, crosslinking with the G blocks to form a uniformly porous gel network [30,33]. However, CaCO_3_ lack the P element essential for osteogenesis [34], and therefore, calcium phosphate low-solubility calcium salts may be a more ideal choice for the preparation of porous hydrogel to effectively promote osteogenic activity [35].

After a comprehensive evaluation, we selected calcium hydrogen phosphate (DCP) as the low-solubility calcium salt for crosslinking sodium alginate. We hypothesized that by combining DCP and GDL, this would facilitate the sustained and endogenous release of Ca^2+^ for the crosslinking of SA, and provide Ca and P elements to enhance the bone repair capability of the hydrogels [36,37,38]. Furthermore, gelatin (GEL), characterized by rapid crosslinking and degradation, was selected as the second phase. The rapidly crosslinked GEL was segregated from the SA phase to form a porous structure [26]. Compared to SA, the swift degradation of gelatin facilitated an increase in pore size within the porous structures during the gradual degradation process. The SA, which degraded slowly in vivo, served to preserve the fundamental morphology of the porous structure.

**Figure 1:**
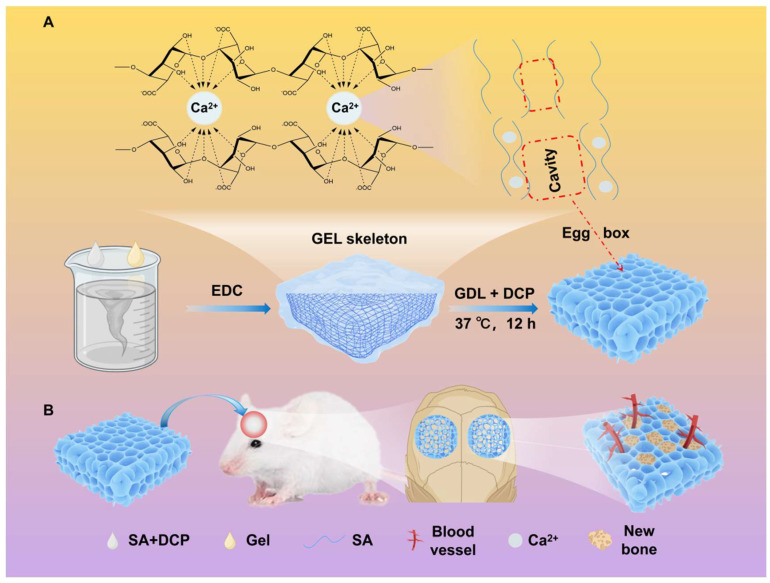
Schematic diagram of (**A**) preparation process of porous hydrogels and formation mechanism of “Egg-box” structure, and (**B**) porous hydrogels promoting vascularized bone regeneration.

In this study, we integrated the “Egg-box” and phase separation methodologies to engineer a double crosslinked SA/DCP/GEL gradual degradable porous hydrogel. This approach utilized a two-step gelation mechanism: (1) initially, gelatin crosslinked rapidly with EDC to form a stable gel framework, (2) followed by the gradual release of Ca^2+^ from DCP with the assistance of GDL, diffusing from the core to the surface, and forming a uniform porous hydrogel structure. Our objective was to facilitate initial cell adhesion through smaller pores early in the implantation process, while larger pores were formed by the gradual degradation at later stages to enhance tissue growth, vascularization, and bone regeneration. A comprehensive investigation was conducted to evaluate the porous architecture, composition, cytocompatibility, and osteogenic activity of these hydrogels, thereby assessing their potential applications in bone repair (Figure 1). In summary, we designed a novel strategy for the preparation of porous hydrogels that exhibit progressively degraded porous structures, which hold potential for enhancing vascularization in bone repair applications.

## 2. Materials and Methods

### 2.1. Materials

Sodium alginate (SA, viscosity 220 ± 20 mPa·s, guluronic acid to mannuronic acid (G/M) ratio of 2), calcium hydrogen phosphate dihydrate (DCP), D-(+)-gluconic acid δ-lactone (GDL, 99%), N-(3-Dimethylaminopropyl)-N-ethylcarbodiimide hydrochloride (EDC, 98.5%), calcium chloride dihydrate (CaCl_2_, ≥99%), and Rhodamine B were purchased from Shanghai Aladdin Biochemical Technology Co., Ltd., Shanghai, China. The gelatin from fish (Gel, 260 Bloom, PI ≈ pH 8.8) was purchased from Shanghai Yuanye Bio-Technology Co., Ltd., Shanghai, China. The newborn calf serum (NBCS) and α-MEM culture medium originated from Gibco (Thermo Fisher Scientific, Waltham, MA, USA). The dexamethasone and β-sodium glycerophosphate were from Shanghai Macklin Biochemical Technology Co., Ltd., Shanghai, China. The Cell Counting Kit-8 (CCK-8), LIVE/DEAD Viability/Cytotoxicity Kit, and BCIP/NBT Alkaline Phosphatase (ALP) color development staining kit were purchased from Dalian Meilun Biological Technology Co., Ltd., Dalian, China.

### 2.2. Preparation of Porous Hydrogel

To verify that the combination of SA-GEL phase separation and the “Egg-box” structure resulted in a uniform mesh porous structure, we conducted a series of experiments. Specifically, 2S5D was used as a control sample, containing only SA, DCP, and GDL, to observe the morphology of the “Egg-box” structure. Meanwhile, 2S5D10G-0GDL, which included SA, DCP, GEL, and EDC, was employed to visualize the porous structure formed by SA-GEL phase separation. Building on this foundation, additional samples—2S5D20G, 2S10D10G, and 2S10D20G—were prepared to investigate the effects of varying DCP and GEL concentrations on the porous structure of the hydrogel. Furthermore, to confirm that the hydrated 2S5D10G hydrogel possessed a porous structure, a dense D-2S5D10G sample crosslinked with CaCl_2_ was used as a control for comparison. According to the proportion of SA, DCP, and GEL as the main components of the hydrogels, the naming abbreviations and component contents of the hydrogels are shown in Table 1.

Figure 1A illustrates the hydrogel preparation process. SA powder was dissolved in deionized water (4% *w*/*v*) at 25 °C, then stirred for 4 h. The gel particles were dissolved in deionized water (20% *w*/*v*, 40% *w*/*v*) at 50 °C and then stirred for 30 min to achieve uniform dispersion. Upon the complete dissolution of sodium alginate, DCP powder (10% *w*/*v*, 20% *w*/*v*) was dispersed into the SA solution to form an SA-DCP suspension. Then, this mixture was stirred for an additional 4 h to ensure the thorough dispersion of the DCP. EDC and GDL (1% *w*/*v*) were dissolved in deionized water and stirred for 5 min to prepare the crosslinking solution. The precursor solutions were prepared by mixing the SA-DCP suspension and the GEL solution at a 1:1 ratio, as detailed in Table 1. The GEL-SA solution was stirred at 37 °C for 30 min, followed by sonicating at the same temperature for an additional 30 min. A total of 1 mL of the crosslinking solution was added to the GEL-SA solution and stirred for 30 s. It is crucial to emphasize that the D-2S5D10G group underwent crosslinking in a 2% CaCl_2_ solution without the inclusion of an additional crosslinking agent to form a dense hydrogel for comparison. The samples were then sonicated for 5 min and stored at 37 °C in an atmospheric pressure incubator for 12 h. During this period, the calcium ions fully diffused and crosslinked with the sodium alginate, resulting in the formation of a porous structure.

### 2.3. Morphological Characterization of the Hydrogels

To characterize the porous structure of the hydrogels, the morphology and structure of the lyophilized hydrogels were observed using scanning electron microscopy (SEM; JSM-6510LV, JEOL, Tokyo, Japan). To prepare the samples for SEM, each sample was washed with PBS, frozen in a −80 °C freezer, and freeze-dried in a lyophilizer [39]. The dry samples were coated with gold prior to SEM observation. Furthermore, micro-CT (VivaCT80, SCANCO Medical AG, Uster, Switzerland) was utilized to conduct 3D reconstruction images of the samples [40]. The scanning parameters were configured at 55 kV, 135 μA, and 8 W, with grayscale thresholds set between 50 and 70. Prior to the experiment, the micro-CT was calibrated in accordance with the vivaCT 80 User’s Guide (SCANCO Medical AG, Switzerland).

ImageJ 1.54 (NIH, Bethesda, MD, USA) was utilized to quantify the hydrogel pore size in both the SEM images and micro-CT images, as well as to determine the porosity from the SEM images [41,42]. Furthermore, data acquired from micro-CT scans were reconstructed using SCANCO Medical Visualizer (SCANCO Medical AG, Switzerland), and the porosity was subsequently calculated.

### 2.4. Compositional Characterization of the Hydrogel

The samples were characterized using X-ray diffraction (XRD, DX-2700BH, Dandong, China) to study their phase composition and crystallinity. The 2θ scanning range for XRD with copper target Kα radiation (λ = 0.1542 nm) was set to 10–80° with a fixed step size of 0.02°. The chemical structures of the samples were characterized at ambient temperature using a Fourier Transform Infrared Spectrometer (FTIR, Nicolet 6700, Waltham, MA, USA) with a spectral resolution of 0.05 cm^−1^ and a wavenumber range from 4000 to 700 cm^−1^. The thermal property of the scaffolds was determined by simultaneous thermal analysis (STA, NETZSCH TG209 F3 Jupiter, Selb, Germany). For STA analysis, measurements were conducted in a N_2_ atmosphere within a temperature range of 30 to 650 °C, at a heating rate of 5 K/min, and with a bulk flow rate of approximately 40 mL/min.

### 2.5. Swelling Test

The freeze-drying weighing method was selected to quantify the swelling ratio of the hydrogel, taking into account that the preservation step during hydrogel preparation may result in water loss [43]. The original mass of freeze-dried hydrogel was recorded as W_0_. After that, the lyophilized hydrogels were studied by immersing them in phosphate-buffer solution (PBS) at 37 °C. At a predetermined time point, the samples were weighed and recorded as W_t_ immediately after blotting water on the surface with absorbent paper. The swelling ratio (SR) of the hydrogels was calculated by Equation (S1) in Supporting Information.

### 2.6. Mechanical Measurement

The compressive strength of the samples was assessed using an electronic universal material testing machine (AG; Instron 5967, Instron, Boston, USA). The hydrogels were fabricated into a cylindrical shape with dimensions of 8 mm in diameter and 10 mm in height, and were equilibrated in PBS for 30 min before undergoing mechanical testing. The scaffolds were loaded with pressure until fracture at a cross-head speed of 1 mm/min. The compressive stresses of the hydrogels were evaluated by the following equation: σ = F/A, where A is the sample cross-section area and F is the applied force. Five samples were measured (*n* = 5).

### 2.7. Degradation Test

To evaluate the in vitro biodegradability, the samples were washed with deionized water, lyophilized, and the dry weight recorded (W0). The dried hydrogel was immersed in collagenase solution (1 μg mL^−1^). At a predetermined time point, the undegraded samples were collected. These collected samples were lyophilized and weighed (Wt). The degradable properties of the hydrogel were evaluated by Equation (S2) in Supporting Information.

### 2.8. Effect of the Scaffolds on BMSC Behaviors

#### 2.8.1. BMSC Culture

Bone marrow mesenchymal stem cells (BMSCs) were isolated from the femurs of 4-week-old female Sprague–Dawley (SD) rats. SD rats (female, approximately 250 g) were procured from Chengdu Dossy Experimental Animals Co., Ltd. (Chengdu, China). All experimental procedures involving animals were conducted in strict adherence to the guidelines reviewed and approved by the Ethics Committee of West China Hospital, Sichuan University (20241113008). The extracted cells were cultured in α-MEM complete medium (α-MEM) containing 10% newborn calf serum (NBCS) and 1% penicillin–streptomycin (P/S), and passages 2–5 were used. The same configuration of α-MEM was used for all cellular experiments with BMSCs. The culture medium was replaced every other day.

#### 2.8.2. Cell Proliferation

The CCK-8 assay was employed to assess the impact of scaffolds on cell proliferation. Samples were prepared as circular thin sections with a diameter of 4 mm and a thickness of 1 mm. These samples were sterilized in ethyl alcohol followed by UV irradiation for 4 h. The BMSCs were seeded on the hydrogels in a 48-well plate at a density of 2 × 10^4^ cells/well and cultured in the incubator for 1, 3, and 5 days, respectively. At a predetermined time point, the medium and CCK-8 stock solution were prepared into the working solution at a volume ratio of 10:1, and 600 μL of CCK-8 working solution was added to each well, which were then placed into an incubator for 90 min. Then, 150 μL of incubation solution was pipetted into a 96-well plate, and the OD values were measured at a wavelength of 450 nm with a microplate reader (PerkinElmer, Waltham, MA, USA).

The cell viability was examined by a Live/Dead assay. After culturing for 7 days, the cells were washed three times with PBS, and then 300 μL mixed staining solution (100 mM Calcein-AM and 200 mM Ethidium Homodimer) was added to the wells containing the samples, which were then incubated for 30 min at room temperature in an incubator. Images of the Live/Dead staining were taken by an inverted biological microscope (ECLIPSE Ti, Nikon, Tokyo, Japan).

#### 2.8.3. Effect of Cell Behavior on Hydrogels

To investigate the impact of cells on the surface and structure of the material after implantation, the BMSCs were seeded on sterile samples in a 24-well plate at a number of 2 × 10^4^ cells per well, and the medium was changed every two days. After culturing for 1 day, 4 days, and 7 days, the samples were washed three times with PBS, lyophilized, and then loaded in SEM to be observed.

#### 2.8.4. Osteogenic Differentiation

The BMSCs were cultured on hydrogels, with each well containing 1 mL of complete α-MEM medium. Once the cells reached 80% confluency in the well plate, the α-MEM medium was substituted with osteogenic induction medium, and the cells were incubated under osteogenic conditions for a duration of 7 days. The osteogenic induction medium was refreshed every two days. After completing the 7-day osteogenic induction period, the cells were washed three times with PBS, fixed with 4% paraformaldehyde for 30 min, and subsequently stained using the ALP Staining Kit for 15–30 min within an incubator. Following staining, the cells were washed three times with PBS, and then observed and photographed under an inverted biological microscope.

#### 2.8.5. Gene Expression

Following a 7-day osteogenic induction of BMSCs, the mRNA expression levels of the target genes, including OCN, ALP, Runx2, and COL1, were measured using reverse transcription quantitative polymerase chain reaction (RT-qPCR). The primer sequences utilized in this study are detailed in Table S1. The ACTB served as the reference gene for normalization.

### 2.9. Subcutaneous Implantation of Hydrogels in Rats

All experimental procedures involving animals were conducted in strict adherence to the guidelines reviewed and approved by the Ethics Committee of West China Hospital, Sichuan University (20241113008). Three-month-old female Sprague–Dawley rats, supplied by Chengdu Dossy Experimental Animals Co., Ltd., were utilized in this study. Prior to the surgical procedures, all animals were anesthetized using isoflurane. Hydrogels (2S5D10G, 2S5D20G) were fabricated as thin sheets with a diameter of 4 mm and a height of 1 mm, swelled with PBS, and sterilized using UV and 75% ethanol. The hydrogels were subcutaneously implanted into each rat, while the D-2S5D10G group served as the control (Ctrl). The rats were humanely euthanized at 7, 14, and 21 days post-surgery. Following euthanasia, tissue samples were promptly collected and fixed in 4% paraformaldehyde (PFA). Three rats at each time point were used to obtain duplicate samples. Hematoxylin and eosin (H&E) staining was performed for preliminary histological analysis. Additionally, the immunohistochemistry (IHC) staining of CD31 and α-SMA (anti-α-SMA, Abcam, Cambridge, UK) was performed to identify blood vessels. The number of vessels were semi-quantitatively analyzed using ImageJ 1.54 [44].

### 2.10. Osteogenesis of Hydrogels

#### 2.10.1. Surgical Procedure

All experimental procedures involving animals were conducted in strict adherence to the guidelines reviewed and approved by the Ethics Committee of West China Hospital, Sichuan University (20241113008). Eight-week-old female Sprague–Dawley rats were utilized to establish a calvarial bone defect model. To evaluate the osteogenesis of the hydrogels, a circular defect was created on both sides of the central suture in the parietal bone using a trephine (ZH-GSZ, Globalebio Technology, Beijing, China) drill with a 4 mm diameter (external diameter) [45,46], after isoflurane anesthesia. The defect area was continuously irrigated with normal saline during the procedure to prevent thermal injury to the surrounding tissues. The 2S5D10G and 2S5D20G hydrogels were prepared and swelled as thin sheets (approximate diameter of 4 mm and height of 1.5 mm). Furthermore, the group in which the same calvarial defect was created but no hydrogel was implanted was named as the Sham group. At each time point, four parallel samples were established for each group. At 4- and 8-weeks post-procedure, the rats were euthanized. The calvarial tissue was then collected and fixed with 4% PFA solution for subsequent analysis.

#### 2.10.2. Micro-CT Analysis

All samples were analyzed using a micro-CT scanner (VivaCT80, SCANCO Medical, Uster, Switzerland). The scanning parameters were configured at 55 kV, 135 μA, and 8 W, with grayscale thresholds set between 212 and 1000. Prior to the analysis, the micro-CT scanner was calibrated in accordance with the vivaCT 80 User’s Guide (SCANCO Medical AG, Switzerland). The acquired micro-CT data were processed using SCANCO Medical Visualizer (SCANCO Medical AG, Switzerland). The regions of interest (ROIs) were defined as circular areas with a diameter of 4 mm, precisely overlapping with the defect regions. Following the reconstruction of the calvarial morphology, the bone volume/total volume (BV/TV, %) ratio, trabecular number (Tb.N, mm^−1^), trabecular thickness (Tb.Th, mm), and trabecular separation (Tb.Sp, mm) were analyzed.

#### 2.10.3. Histological Analysis

Paraffin blocks were prepared for histological analysis. Hematoxylin and eosin (H&E) staining and Masson trichrome staining were performed on the tissue sections to assess the histological morphologies. Immunofluorescence (IF) staining was conducted using anti-CD31 antibody (Abcam, Cambridge, UK) to specifically label the blood vessels. The density of the vessels was semi-quantitatively analyzed using ImageJ 1.54.

### 2.11. Statistical Analysis

Data are presented as the means ± SD. Statistical analysis was performed using one-way analysis of variance (ANOVA) with Tukey’s posttest using IBM SPSS Statistics Version 23.0 (SPSS 23, IBM Corporation, Armonk, New York, NY, USA). * *p* < 0.05, ** *p* < 0.01, and *** *p* < 0.001 were considered to be statistically significant.

## 3. Results and Discussion

### 3.1. Morphology and Three-Dimensional Structure of Porous Hydrogels

The specific formation mechanism is illustrated in Figure 1A. EDC-mediated rapid crosslinking of GEL triggered the spatial segregation of uncrosslinked SA within the gelatin network. Over a period of 12 h, the acidification of GDL enabled sustained Ca^2+^ release, progressively inducing the crosslinking of SA within the gelatin network. Concomitant with SA crosslinking progression, significant phase separation between SA and GEL ensued, resulting in the formation of a mesh-like porous structure under the synergistic effect of the GEL network and the “Egg-box” structure of SA. To validate the dual contributions of SA-GEL phase separation and alginate “Egg-box” assembly to the hydrogel porosity, controlled experiments and comparative analysis were conducted with the 2S5D, 2S5D10G-0GDL, and 2S5D10G groups. As illustrated in Figure S2A, the 2S5D control exhibited a porous structure through the “Egg-box”, with SA and DCP serving as the matrix materials. However, a marked non-uniform structure was observed, potentially attributable to the formation of a compact layer by Ca^2+^ and SA, which hindered further Ca^2+^ diffusion [47]. To address this issue, GEL was incorporated to induce phase separation from SA, leading to the design of the 2S5D10G-0GDL sample aimed at improving the porous structure. In the 2S5D10G-0GDL sample, GDL was removed to minimize Ca^2+^ release, ensuring that the porous structure was primarily formed by the phase separation of SA and GEL. As illustrated in Figure S2B, the 2S5D10G-0GDL group exhibited a distinct macroporous structure. However, its morphology remained non-uniform, likely due to the stochastic nature of SA-GEL phase separation. To improve the uniformity of the porous structure of materials, we integrated the advantages of two strategies: (1) exploiting the rapid crosslinking property of GEL to form a more uniform network structure, and (2) using time-dependent Ca^2+^ liberation to potentiate SA “Egg-box” formation and subsequent phase separation. Consequently, by combining these two approaches, the 2S5D10G sample was designed, resulting in a relatively uniform porous structure.

**Figure 2:**
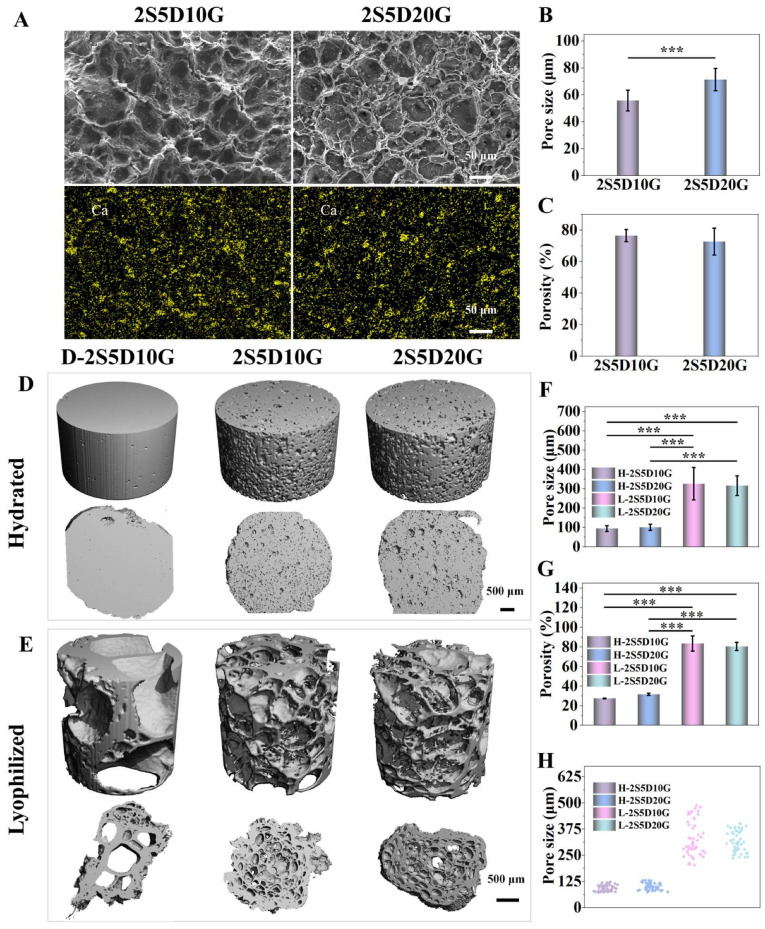
Porous structure of the hydrogels. (**A**) SEM images of the porous hydrogels and EDS mapping of the calcium (Ca, yellow). (**B**) The average pore size and (**C**) the porosity of the hydrogels (SEM images). The micro-CT images of (**D**) the hydrated hydrogel and (**E**) the lyophilized hydrogel. (**F**) The pore size of the hydrated and lyophilized hydrogels (micro-CT images). (**G**) The porosity and (**H**) the pore size distribution of the hydrated and lyophilized hydrogels (micro-CT images) (*n* = 50) (*** *p* < 0.001; one-way ANOVA with Tukey’s posttest).

The contents of calcium hydrogen phosphate and gelatin were screened and optimized. Four different groups of composite hydrogels were considered, namely, 2S5D10G, 2S5D20G, 2S10D10G, and 2S10D20G. The structural characteristics of the sol before and after crosslinking with EDC and Ca^2+^ are illustrated in Figure S1. Through the action of EDC and Ca^2+^, the sol (Figure S1A) was transformed into a gel (Figure S1B). The morphology of each hydrogel group was observed using SEM (Figure 2A and Figure S2). As illustrated in Figure 2A, the 2S5D10G and 2S5D20G groups exhibited a uniform porous structure. However, the formation of pores in the 2S10D10G group was not uniform (Figure S2C). Although the 2S10D20G group retained a porous structure, no obvious interconnective pores were observed and a large amount of DCP precipitation was presented (Figure S2D). This implies that the increase in the content of either DCP or GEL was not favorable for the construction of porous hydrogel networks. This may be due to the fact that the high percentage of DCP precipitated would continue to accumulate on the pore walls and the gelatin could not maintain its original morphology, leading to the collapse of the porous structure in the 2S10D10G group. The high proportion of gelatin led to the thickening of the pore wall of the 2S10D20G group, which prevented the interconnection of the pores while maintaining the porous structure. Therefore, the 2S5D10G and 2S5D20G groups, which possessed an excellent microporous morphology, were selected for the subsequent study. Semi-quantitative statistical analysis from the SEM images showed that the average pore diameters of the 2S5D10G and 2S5D20G groups were 55.77 ± 7.75 μm and 71.34 ± 8.26 μm, respectively, and the average porosities were 76.55 ± 3.85% and 72.68 ± 8.55%, respectively (Figure 2B,C). The above results confirmed the successful crosslinking of sodium alginate-based hydrogels using calcium hydrogen phosphate and gelatin to construct the expected porous hydrogels.

Considering that the microporous structures of the hydrogels observed by SEM after freeze-drying had a large difference compared to the swelling state and could not accurately reflect the porous structure of the hydrogels in the microenvironment of osteogenesis, the micro-CT 3D reconstructions of the 2S5D10G and 2S5D20G hydrated hydrogels were performed, and the hydrogel crosslinked with CaCl_2_ (D-2S5D10G) was used as a control (Ctrl). The microporous structures of the 2S5D10G and 2S5D20G groups were further analyzed by micro-CT 3D reconstruction in the wet and lyophilized states, respectively. As expected, the pore size and porosity of the 2S5D10G and 2S5D20G hydrogel groups in the wet and lyophilized states were quantitatively significantly different (Figure 2D–H). With the 2S5D10G group as the representative, the pore size ranged from 72.63 μm to 123.59 μm in the wet state and 203.91 μm to 487.89 μm in the lyophilized state, respectively. Notably, the hydrogel crosslinked with calcium chloride showed a dense structure in the wet state. In contrast, the 2S5D10G and 2S5D20G hydrogels had a porous structure in the wet state, which facilitated cell penetration into the hydrogels and nutrient transfer after implantation, and thus they may better promote bone tissue regeneration.

### 3.2. Physicochemical Properties of Porous Hydrogels

**Figure 3:**
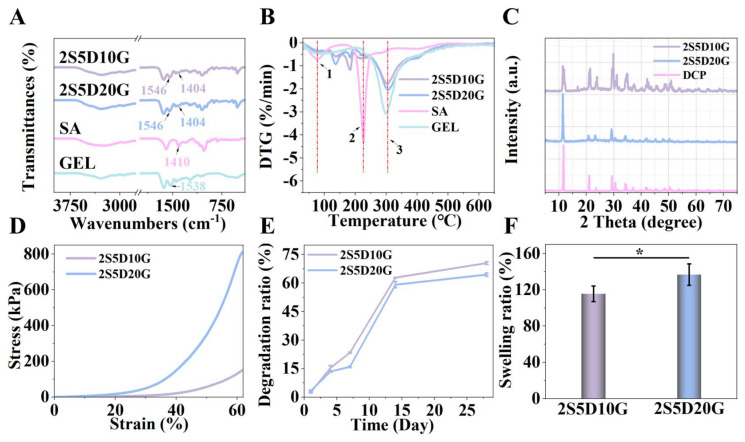
(**A**) FTIR spectra and (**B**) DTG curves of SA, GEL, 2S5D10G, and 2S5D20G. (**C**) XRD patterns of 2S5D10G, 2S5D20G, and DCP. (**D**) Stress–strain curves of 2S5D10G and 2S5D20G hydrogels. (**E**) Degradation and (**F**) swelling ratio of 2S5D10G and 2S5D20G hydrogels (*n* = 3) (* *p* < 0.05; one-way ANOVA with Tukey’s posttest).

Figure 3A presents the FTIR spectra of 2S5D10G, 2S5D20G, SA, and GEL. All spectra exhibit broad peaks in the range of 3000–4000 cm^−1^, which are attributed to O-H stretching vibrations. The peak at 1410 cm^−1^ for SA is assigned to the symmetric stretching vibrations of the -COO-. Notably, the characteristic peak of -COO- of SA shifts from 1410 cm^−1^ to 1404 cm^−1^ in 2S5D10G and 2S5D20G, indicating an interaction between the -COO- and Ca^2+^ [48]. Furthermore, the spectrum of GEL shows characteristic absorption peaks at 1538 cm^−1^, corresponding to amide II. Following the crosslinking of GEL with EDC, the characteristic peak shifts from 1538 cm^−1^ to 1546 cm^−1^ in 2S5D10G and 2S5D20G. This may be attributed to the carboxyl group of aspartic acid in GEL being activated when using EDC, which then facilitates the formation of an isopeptide bond with the amino group [49]. These FTIR results confirm the interactions between SA and Ca^2+^, as well as between GEL and EDC. As shown in Figure 3B, the DTG curves of SA, GEL, 2S5D10G, and 2S5D20G exhibit three similar weight loss peaks. Peaks 1 and 2 correspond to water loss, cleavage into intermediates, and the carbonization process of SA, respectively, while peak 3 is associated with the cleavage and degradation of GEL. Compared to the DTG curves of SA and GEL, the positions and temperatures of the weight loss peaks for 2S5D10G and 2S5D20G show no significant changes, indicating that the SA and GEL in the hydrogel exist as separate phases [50]. In conjunction with the alterations in the pores of the 2S5D and 2S5D10G, this finding further substantiates that the phase separation of SA and GEL facilitates the development of porous structures.

Given that calcium and phosphorus exist in various forms, the specific types of calcium and phosphorus salts in the hydrogels are identified by XRD. XRD analysis indicates that DCP is the predominant form of calcium and phosphorus in both the 2S5D10G and 2S5D20G samples (Figure 3C). Compared to hydroxyapatite (HA), which exhibits high crystallinity and stability, DCP demonstrates faster in vivo degradation characteristics [51]. This enhancement will contribute to improving the osteogenic properties of hydrogels [52,53].

In addition, it has been established that the mechanical behavior of materials influences cell proliferation and differentiation [54]. As shown in Figure 3D, the compressive deformation of the two hydrogels exceeds 60%, and the breaking strengths of 2S5D10G and 2S5D20G exceed 150 kpa and 750kpa, respectively, indicating that these materials possess satisfactory mechanical properties. It is noteworthy that the 2S5D10G and 2S5D20G hydrogels maintain structural integrity during compression, without any instances of fracture. This exceptional performance can be attributed to the porous structure providing a buffer space for deformation within the material, while simultaneously benefiting from the dual network skeleton composed of SA and GEL which absorbs energy to maintain structural stability.

An appropriate degradation rate of the hydrogel is beneficial for providing sufficient support for bone development [5]. The degradation behavior of hydrogels is characterized by mass loss and mechanical disintegration [55]. The degradation experiment results demonstrate that both the 2S5D10G and 2S5D20G groups exhibit a degradation time exceeding 4 weeks, with approximately 70% at the end of the fourth week (Figure 3E). The 2S5D20G hydrogel contains a higher concentration of GEL, exhibiting a slower degradation rate compared to the 2S5D10G group. The swelling rates for the 2S5D10G and 2S5D20G groups are 115.59 ± 8.68% and 136.73 ± 11.83%, respectively (Figure 3F). The highly swellable GEL network and the low-swelling SA network may contribute to this phenomenon [56]. The swellable GEL network enables the hydrogel to conform closely to the repair site. The low-swellable SA network preserves the structural integrity of the hydrogel and ensures the functionality of its porous architecture.

The morphology and physicochemical properties of the hydrogels demonstrate that the objective of combining the “Egg-box” model with phase separation is successfully achieved through the two-step gelation method, resulting in the preparation of uniformly porous hydrogels. Specifically, except for the porous structure which facilitates cell migration, nutrient and waste exchange, tissue growth, and vascularization, DCP may be beneficial to enhancing the osteogenic properties of the hydrogels.

### 3.3. Cytocompatibility of Porous Hydrogels

**Figure 4:**
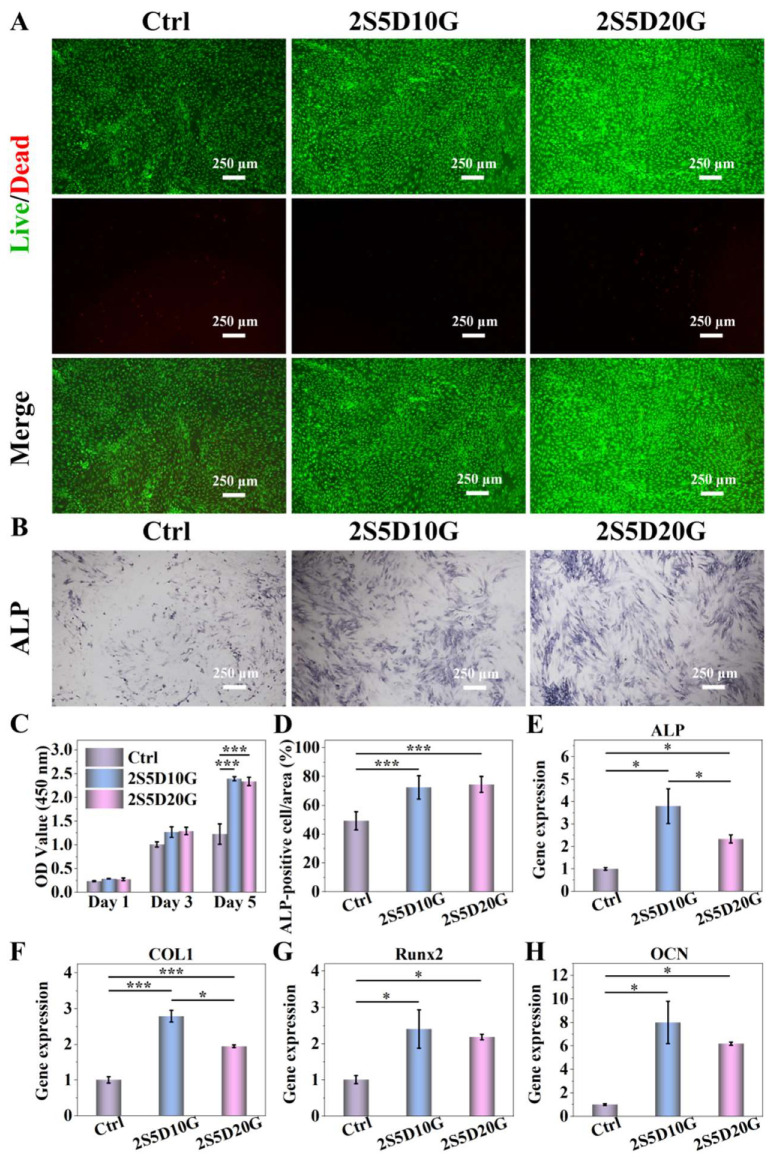
(**A**) Live/Dead fluorescence images of BMSCs cultured on porous hydrogels for 7 days. (**B**) Representative images and (**D**) semi-quantitative analysis of ALP staining of BMSCs after 7 days of culture. (**C**) CCK-8 test of BMSCs cultured on porous hydrogels for 1, 3, and 5 days. (**E**–**H**) ALP, COL1, Runx2, and OCN expression levels after BMSC incubation for 7 days (*n* = 3) (* *p* < 0.05 and *** *p* < 0.001; one-way ANOVA with Tukey’s posttest).

The biocompatibility of hydrogels is crucial for bone repair applications [57]. Live/Dead staining experiments revealed that compared to the Ctrl group, a small number of dead cells (red) and a large number of living cells (green) were observed in the hydrogel groups. Furthermore, these living cells exhibited excellent morphological characteristics, indicating that both porous hydrogels showed good cytocompatibility (Figure 4A). The cell proliferation activity of BMSCs cultured on the surface of 2S5D10G and 2S5D20G hydrogels set for 1, 3 and 5 days were tested by CCK-8 assay, as shown in Figure 4C. There was no significant difference in the proliferative activity of the cells in each group after 1 day of culture. When cultured for 3 and 5 days, the proliferative activity of BMSCs in both the 2S5D10G and 2S5D20G hydrogel groups was higher than that of the control group. This suggests that the hydrogels exhibited excellent cellular compatibility, and their porous structure was likely a significant factor in promoting cell proliferation and infiltration [58].

**Figure 5:**
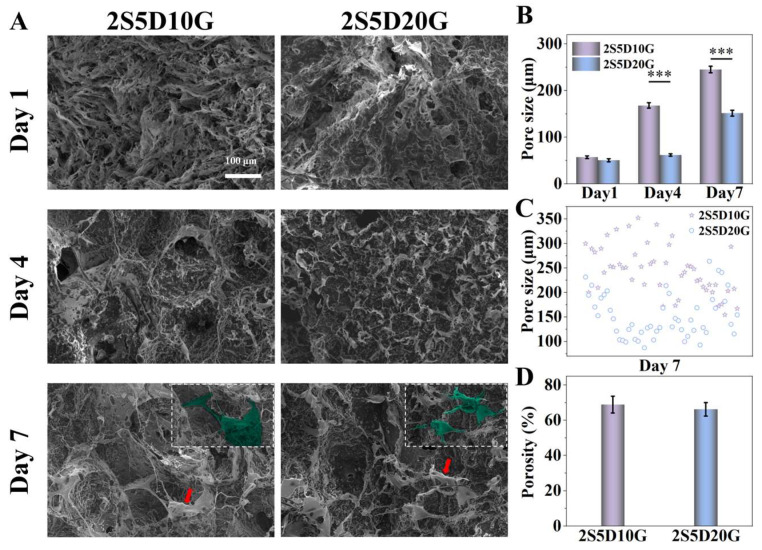
(**A**) SEM images of hydrogels and cells following the co-culture. (**B**) The average pore size of hydrogels. (**C**,**D**) The pore size distribution and porosity of hydrogels after 7 days of co-culture. The red arrows indicate cells that have been assigned pseudo colors for visualization purposes (*n* = 3) (*** *p* < 0.001; one-way ANOVA with Tukey’s posttest).

As a significant mineralizing enzyme in the process of bone formation and regeneration, ALP can decompose organic phosphorus and produce inorganic phosphorus, which promotes mineral deposition and hydroxyapatite formation, and is an important indicator of the proteogenic differentiation of osteoblasts [59,60]. The expression of ALP activity was determined by co-culturing the cells with porous hydrogels for 7 days. Qualitative and quantitative analysis of ALP showed that the 2S5D10G and 2S5D20G porous hydrogel groups had higher positive expression compared to the control group (Figure 4B,D). The mRNA expression of four osteogenesis-related genes, ALP, COL1, Runx2, and OCN, was determined after inoculating BMSCs onto the surface of the porous hydrogels for 7 days. As shown in Figure 4E,F, the expression of the four genes was higher in the 2S5D10G and 2S5D20G porous hydrogel groups than in the control group. Meanwhile, the highest expression of ALP and COL1 was found in the 2S5D10G group, which was significantly higher than that in the 2S5D20G group. The rapid degradation of DCP within the hydrogels facilitated the release of Ca and P elements, thereby promoting the osteogenic activity expression of BMSCs. Notably, the 2S5D10G hydrogel exhibited the most significant positive effect. This may be attributed to the higher gelatin content and slower degradation rate of the 2S5D20G hydrogel (Figure 5), which hindered the release and degradation of the embedded DCP, leading to reduced osteogenic activity compared to the 2S5D10G group.

### 3.4. The Degradation of Porous Hydrogels in the Cellular Environment

The degradation of the scaffold plays a crucial role in the process of bone repair, which can provide temporary support and facilitate revascularization to improve the therapeutic efficacy, and is a key factor in achieving the effective repair of bone defects [61]. Therefore, it is crucial to consider the potential degradation of porous structures during the disintegration process of porous hydrogels [55]. The degradation of the hydrogel in the biological microenvironment was observed by co-culturing porous hydrogel with BMSCs at different time points. As shown in Figure 5B, from the first day to the seventh day of co-culture, the pore size of the hydrogel gradually increased, showing a desired hydrogel degradation performance. With the extension of the co-culture time, the gelatin in the hydrogel was degraded gradually (Figure 5A). As a result, semi-quantitative statistical analysis from SEM images showed that the pore size of the 2S5D10G group gradually increased to 168.23 ± 5.84 μm at 4 days and reached 250~300 μm at 7 days (Figure 5B). This indicated that the porous structure of the hydrogel not only remained integral after seven days of degradation but also expanded into a configuration more conducive to osteogenesis. The slower alteration in pore size observed in the 2S5D20G group compared to the 2S5D10G group can likely be attributed to the increased thickness of the pore walls and the consequently decelerated degradation rate, which was the result of the higher GEL content in the 2S5D20G samples. Similar phenomena have also been reported in the earlier literature [62]. After the 7 days of co-culture, the pore sizes of the 2S5D10G group ranged from 200 to 350 μm, while those of the 2S5D20G group ranged from 100 to 250 μm (Figure 5C). Furthermore, after 7 days of co-culture, semi-quantitative statistical analysis from SEM images showed that the porosities of 2S5D10G and 2S5D20G were measured at 68.88 ± 4.80% and 66.19 ± 3.81%, respectively. This structure met the requirements for the pore architecture during tissue regeneration, thereby promoting tissue growth and vascularization and ultimately enhancing bone tissue regeneration [63,64,65]. Notably, at 7 days of co-culture, significant cell infiltration could be observed in the porous structure of the hydrogels. In conclusion, the porous hydrogel possesses the desired degradation properties and presents a gradual evolution of pore structure in the biological microenvironment. The small initial pore size of hydrogel scaffolds can provide more sites for cell adhesion, and the gradual degradation to form large pores is conducive to cell growth and blood vessel formation, and ultimately conducive to functional bone regeneration.

### 3.5. Subcutaneous Implantation of Porous Hydrogels in Rats

**Figure 6:**
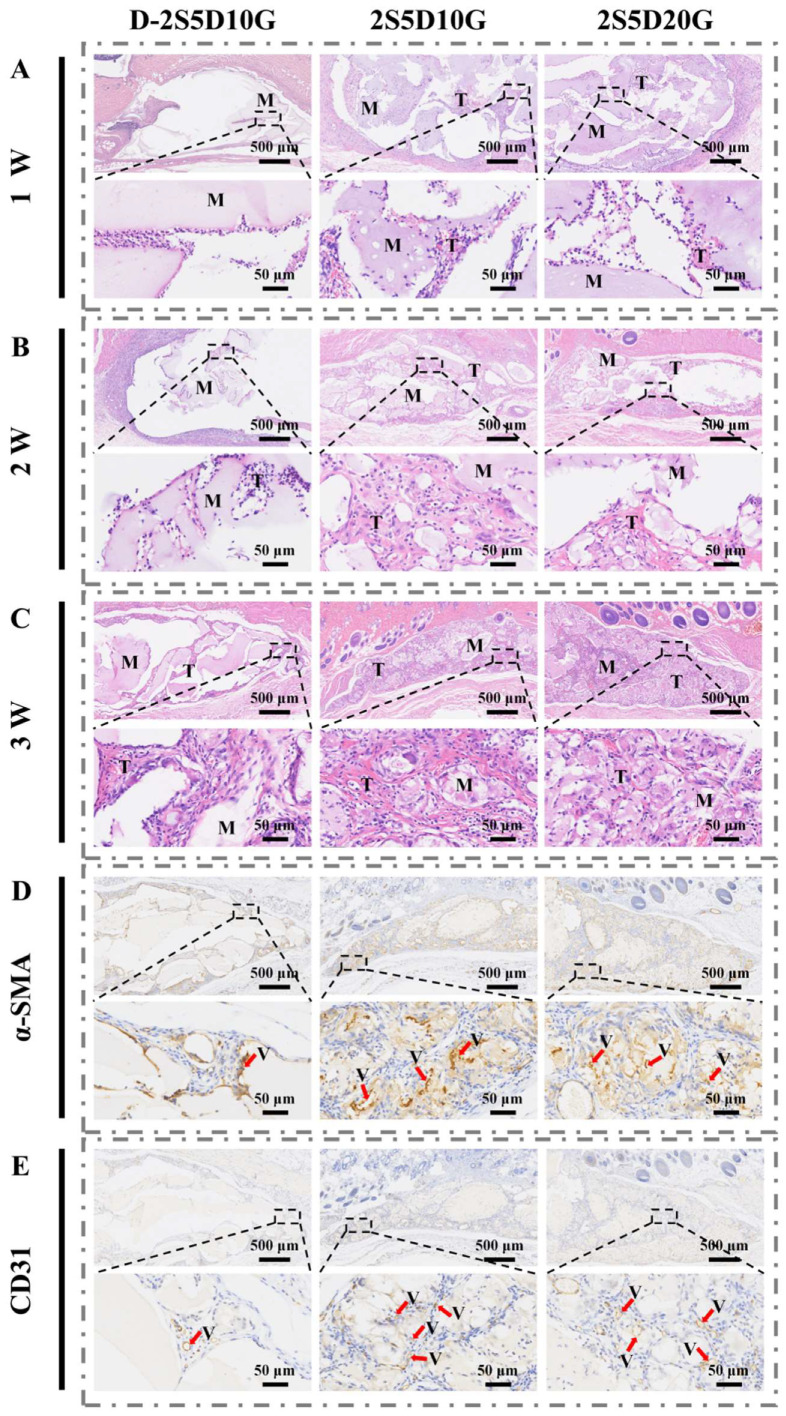
Histological analysis was used to analyze the cell infiltration, tissue growth, and angiogenesis after hydrogel implantation in rats subcutaneously. (**A**–**C**) H&E staining of the hydrogels implanted after 1, 2, and 3 weeks. Representative image of immunohistochemical staining for (**D**) α-SMA and (**E**) CD31 at 3 weeks. The red arrows indicate the positive regions of α-SMA and CD31, “M” denotes hydrogel materials, “T” represents tissue, “V” indicates blood vessels, and “W” means week.

Subcutaneous implantation is a key technique to assess cell infiltration and tissue growth [66,67]. The D-2S5D10G, 2S5D10G, and 2S5D20G hydrogels were used for subcutaneous implantation in rats for a period of 1, 2, and 3 weeks to assess the cell infiltration and tissue growth of porous hydrogels in vivo. Comparative analyses of subcutaneous implantation at different time points showed the gradual infiltration of cells and tissues from the surrounding environment into the hydrogel matrix. As shown in Figure 6A, one week after implantation, the 2S5D10G and 2S5D20G groups showed significant cellular infiltration with minimal tissue formation, while the dense D-2S5D10G hydrogel group showed only limited cellular infiltration. The experimental results at 2 weeks after implantation also confirmed this finding (Figure 6B). More importantly, both the 2S5D10G and 2S5D20G groups exhibited complete cellular infiltration and full tissue integration within the hydrogel matrix after 3 weeks of implantation, in contrast to the dense D-2S5D10G group (Figure 6C). In conclusion, the porous 2S5D10G and 2S5D20G groups performed better in promoting cellular infiltration and tissue growth compared to the dense hydrogels.

α-SMA and CD31 staining were utilized to quantify the number of newly formed blood vessels in the subcutaneous implantation region [68,69]. Blood vessels with brown-stained cytoplasm were classified as positive. As illustrated in Figure 6D,E and Figure S3, the number of vessels in the 2S5D10G and 2S5D20G groups were 11.44 ± 2.60 and 10.89 ± 1.96, respectively, significantly higher than the 1.56 ± 0.53 observed in the D-2S5D10G group. These findings suggest that the porous hydrogel exhibits a robust capability to promote angiogenesis.

### 3.6. Repair of Rat Cranial Defects with Porous Hydrogels

**Figure 7:**
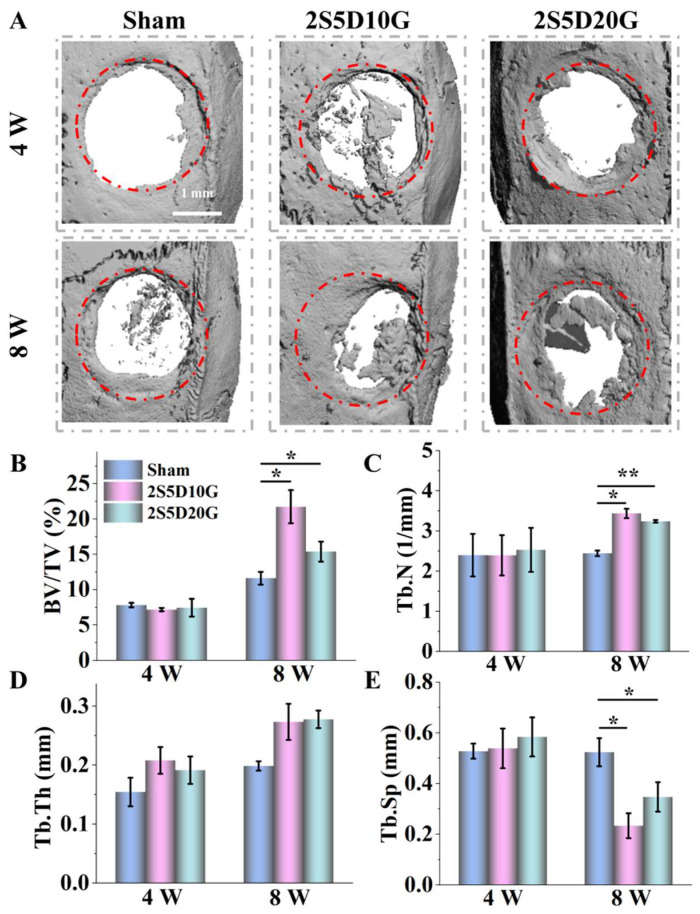
Micro-CT analysis of bone regeneration mediated by porous hydrogels in a rat critical-sized cranial bone defect model. (**A**) The 3D micro-CT images of the rat skull defect after 4 and 8 weeks of treatment. (**B**–**E**) BV/TV, Tb.N, Tb.Th, and Tb.Sp of the Sham, 2S5D10G, and 2S5D20G groups (*n* = 3). The red circle indicates the initial range of the calvarial defect (* *p* < 0.05 and ** *p* < 0.01; one-way ANOVA with Tukey’s posttest).

The in vivo angiogenic and osteogenic properties of the porous hydrogel were evaluated by a cranial bone defect repair model in SD rats. The procedure of hydrogel implantation is shown in Figure S5. By the fourth week of the experiment, micro-CT scans and 3D reconstruction analysis showed that new bone in both the 2S5D10G and 2S5D20G groups extended from the periphery to the center of the defect, whereas the Sham group showed only slight new bone formation at the edges of the defect (Figure 7A). This trend became more pronounced with time. Eight weeks after implantation, new bone formation increased significantly in the 2S5D10G and 2S5D20G groups. The bone volume/tissue volume (BV/TV) was higher in the 2S5D10G and 2S5D20G groups compared to the Sham group. The BV/TV value in the 2S5D10G group even exceeded 20%, which was higher than that in the 2S5D20G group (Figure 7B). In addition, the changes in the trabecular number (Tb.N), trabecular thickness (Tb.Th), and trabecular distance (Tb.Sp) in the 2S5D10G and 2S5D20G groups were consistent with those of BV/TV, while the changes in these indexes in the Sham group were not significant (Figure 7C–E). The above micro-CT results indicate that porous hydrogels can effectively promote bone regeneration, especially the 2S5D10G group, which showed the greatest osteogenic potential.

**Figure 8:**
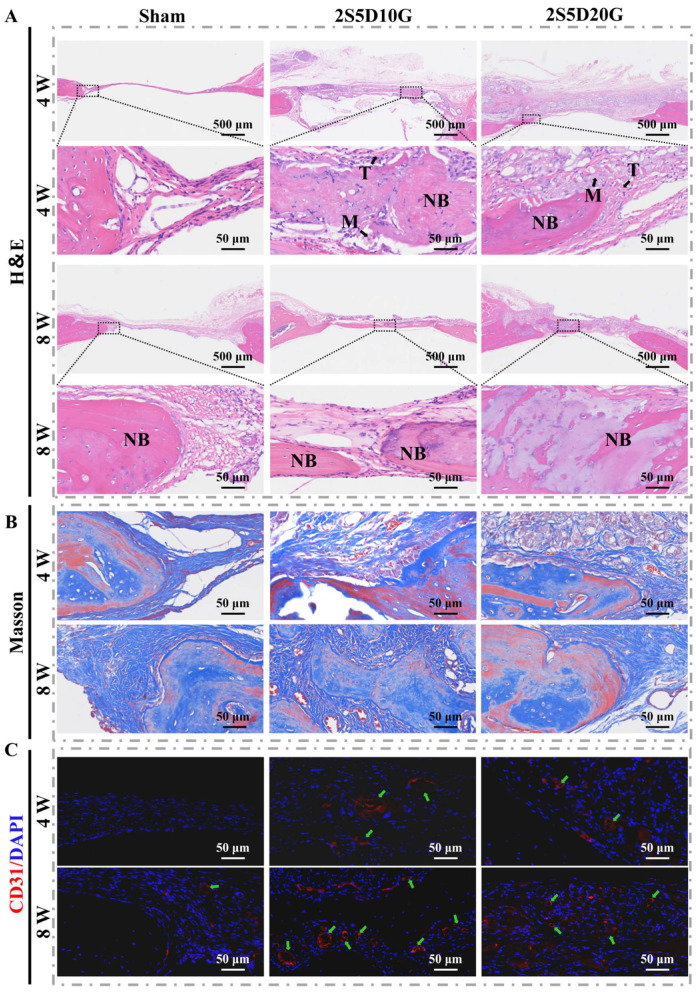
(**A**) H&E and (**B**) Masson staining of the Sham, 2S5D10G, and 2S5D20G groups. (**C**) IF staining images of bone tissue in the Sham, 2S5D10G, and 2S5D20G groups. “M” indicates hydrogel materials, “NB” denotes new bone tissue, and the green arrow indicates blood vessels.

H&E and Masson staining were further used to assess vascularization and new bone formation in the defect area (Figure 8A,B). As shown in Figure 8A, four weeks post-surgery, the defect area in the Sham group was predominantly filled with fibrous tissue, with only a small amount of new bone tissue observed at the periphery. Notably, in the 2S5D10G group, substantial new bone tissue formation was observed within the defect area, accompanied by neatly arranged osteoblasts at the margins. Eight weeks post-operation, the Sham group showed increased bone tissue within the defect area, while fibrous tissue remained the predominant component. In the 2S5D20G group, there was marked inward growth of bone tissue from the defect margins, with an observable transformation from woven bone to lamellar bone within the defect. Notably, in the 2S5D10G group, extensive new bone growth extended inward from the periphery, forming a bridge at the center of the defect, with a similar woven-to-lamellar bone transformation. These findings suggest that the porous hydrogels in both the 2S5D10G and 2S5D20G groups provided favorable conditions for cell adhesion, infiltration, and tissue ingrowth, with the 2S5D10G group demonstrating superior osteogenic effects.

As shown in Figure 8B, newly formed collagen fibers, stained blue, were observed surrounding the newly formed bone within the defect area after 4 weeks of implantation. Both the 2S5D10G and 2S5D20G groups exhibited collagen deposition and angiogenesis within the hydrogel. After 8 weeks of implantation, the majority of the hydrogel in both the 2S5D10G and 2S5D20G groups had degraded, with a substantial amount of new collagen filling the defect area. The presence of osteoblasts and collagen deposition in these groups suggests the sustained osteogenic activity of the porous hydrogels over the long term.

Angiogenesis is fundamental to the process of bone repair, with the CD31 protein serving as an essential component in this mechanism [70,71]. To further evaluate the impact of porous hydrogel scaffolds on vascularized bone regeneration, the vascularization effect of the hydrogel was analyzed using immunofluorescence staining. As shown in Figure 8C, CD31 expression was significantly higher in the 2S5D10G and 2S5D20G groups compared to the Sham group. The semi-quantitative analysis of the number of blood vessels (Figure S4) further indicated that the 2S5D10G and 2S5D20G groups had a high vascularization effect, and 2S5D10G had the strongest vascularization activity at eight weeks.

Overall, both 2S5D10G and 2S5D20G exhibited superior osteogenic properties compared to the Sham group, attributed to the gradual porous structure of the hydrogel and the osteoinductive capability of DCP. Notably, 2S5D10G demonstrated a more pronounced evolution in its porous structure during degradation, with its pore sizes increasing from approximately 60 μm to about 250 μm within one to seven days of degradation. This structural transformation not only enhanced initial cell adhesion upon hydrogel implantation but also facilitated subsequent tissue growth and vascularization, ultimately promoting vascularized bone regeneration [13,14,15,16,17]. Additionally, the accelerated degradation rate of 2S5D10G may have led to a greater release of DCP, which stimulated the osteogenic activity expression of BMSCs and further potentiated osteogenesis in the defect area.

## 4. Conclusions

The SA/DCP/GEL porous composite hydrogels were successfully developed through a two-step gelation process. The fabricated porous hydrogels initially featured small pores, and gradually evolved to large pores during the gradient degradation in the cellular microenvironment. The in vitro and in vivo biological experiments confirmed that the composite porous hydrogels exhibited capabilities in promoting cell proliferation, infiltration, osteogenic differentiation, and vascular growth, consequently promoting vascularized bone regeneration. These findings substantiate the potential application value of our proposed strategy for the preparation of porous hydrogel to promote vascularized bone regeneration.

## Figures and Tables

**Table 1:** Name and composition of hydrogels.

	Content	SA (% *w*/*v*)	DCP (% *w*/*v*)	GEL (% *w*/*v*)	GDL (% *w*/*v*)	EDC (% *w*/*v*)
Name						
2S5D10G		2	5	10	1	1
2S5D20G		2	5	20	1	1
2S10D10G		2	10	10	1	1
2S10D20G		2	10	20	1	1
D-2S5D10G		2	5	10	0	0
2S5D		2	5	0	1	0
2S5D10G-0GDL		2	5	10	0	1

## Data Availability

The data presented in this study are available upon reasonable request from the corresponding author.

## Supplementary Materials

The following supporting information can be downloaded at: https://www.mdpi.com/article/10.3390/jfb16030100/s1, Figure S1: (A) The pictures of the precursor solutions. (B) The pictures of the hydrogels; Figure S2: The SEM images of 2S5D, 2S5D10G-0GDL, 2S10D10G and 2S10D20G hydrogels; Figure S3: Semi-quantitative analysis of vessels number in subcutaneous implantation at 3 weeks; Figure S4: Semi-quantitative analysis of vessels number in new bone; Figure S5: (A) The image of the calvarial defects model. (B) The image of a calvarial defects model following hydrogels implantation. (C) Post-suturing appearance of the calvarial skin; Table S1; Equation (S1); Equation (S2).
