# Supplementary material for: Porous Hydrogels Prepared by Two-Step Gelation Method for Bone Regeneration
Source: J Funct Biomater. 2025 Mar 13;16(3):100. doi: 10.3390/jfb16030100 (PMC11942705; doi:10.3390/jfb16030100)
Supplement: Supplementary file 1 [file jfb-16-00100-s001.zip › jfb-3493395-supplementary.pdf]

## Supporting Information

# Porous Hydrogels Prepared by Two-Step Gelation Method for Bone Regeneration

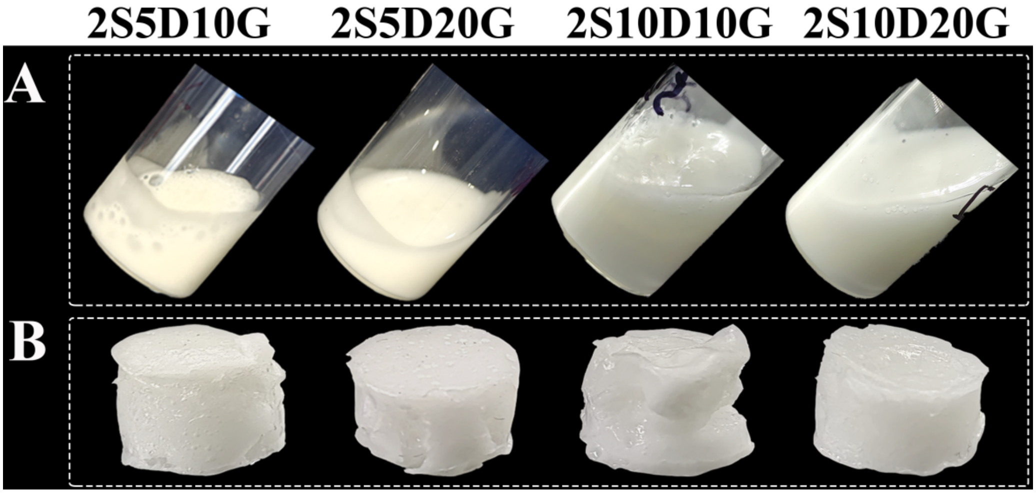


Figure S1 A) The pictures of the precursor solutions. B) The pictures of the hydrogels.

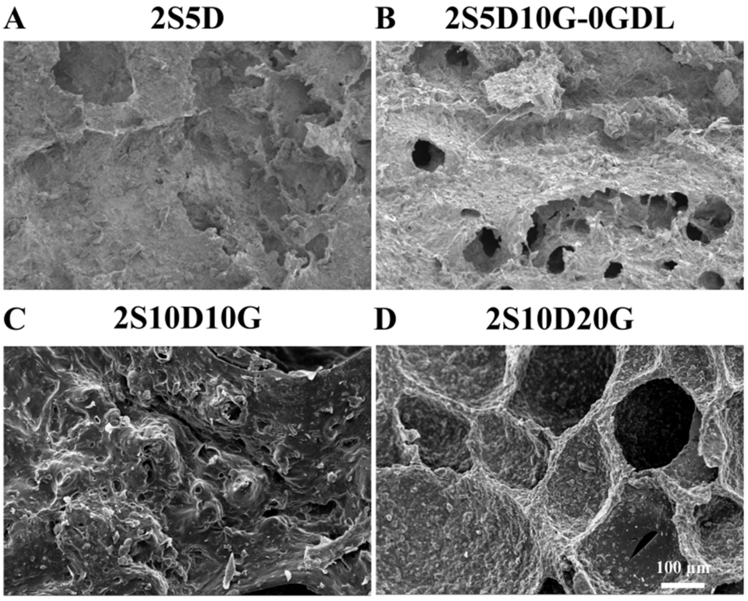


Figure S2 The SEM images of 2S5D, 2S5D10G-0GDL, 2S10D10G and 2S10D20G hydrogels.

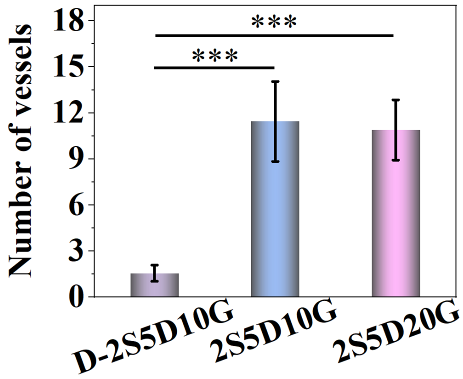


Figure S3 Semi-quantitative analysis of vessels number in subcutaneous implantation at 3 weeks. (***$p < 0.001$; one-way ANOVA with Tukey's posttest).

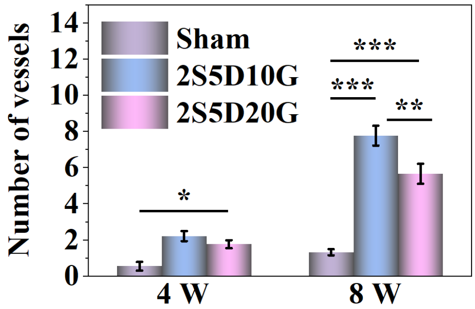


Figure S4 Semi-quantitative analysis of vessels number in new bone. (*$p < 0.05$, **$p < 0.01$, and ***$p < 0.001$; One-way ANOVA with Tukey posttest).

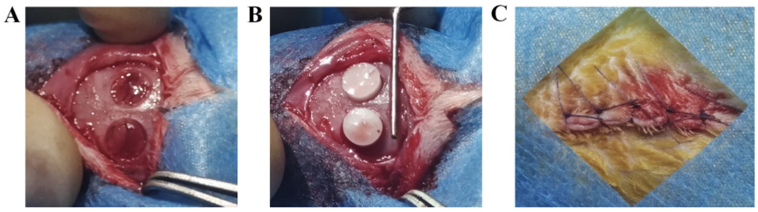


Figure S5 (A) The image of the calvarial defects model. (B) The image of a calvarial defects model following hydrogels implantation. (C) Post-suturing appearance of the calvarial skin.

Table S1

| **Target DNA** | **Primer sequence (5'-3')** |
|---|---|
| *Runx-2* | F:CAGTATGAGAGTAGGTGTCCCGC<br>R:AAGAGGGGTAAGACTGGTCATAGG |
| *ALP* | F:CGGCACCTGCCTTACCAACT<br>R:ACTGTGGAGACGCCCATACC |
| *OCN* | F:GGACCCTCTCTCTGCTCACTCTG<br>R:ACCTTACTGCCCTCCTGCTTGG |
| *COL1* | F:CCCAGCGGTGGTTATGACTT<br>R:TCGATCCAGTACTCTCCGCT |

Equation S1

$$\mathrm{SR}\ (\%) = \frac{W_t - W_0}{W_0} \times 100\% \qquad \text{(S1)}$$

Equation S2

$$\text{Degradation Rate}\ (\%) = \frac{W_t - W_0}{W_0} \times 100\% \qquad \text{(S2)}$$
